# Ammonia vs. Zinc Fillings

**Published:** 1888-01

**Authors:** Levitt E. Custer

**Affiliations:** Springfield


### Ammonia vs. Zinc Fillings.

BY LEVITT E. CUSTER, B. S., D. D. S., SPRINGFIELD.

[Read before the Ohio State Dental Society, Springfield, Ohio, October, 1887.]

The destruction of zinc fillings has usually been attributed to attrition and the "oral fluids." The modus operandi of the former is easily understood, while that of the latter is more vague of comprehension. It is easy to understand why a phosphate cusp should gradually disappear, but why, in some cases, disintegration of the same filling takes place at and under the gum we cannot so easily answer. Often there are cases where the subgingival destruction exceeds that at the more prominent points. The surface produced by wear is hard and more or less smooth, while that produced by the oral fluids seems to be porous and under process of disintegration.

The oral fluid is often blamed and made to cover a vast amount of ignorance because of its complex nature, but I shall try to prove that it is not an oral fluid as such at all which produces this disintegrating effect so often found at the necks of the teeth. In one instance the presence of some one distinct agent is acknowledged, for it has been recommended to fill the cervical border of the cavity with gutta-percha and complete with phosphate thus avoiding the force of this agent. If the teeth are bathed in the oral fluids entirely, why this peculiar disintegration at just one point? The observing dentist upon reflection will acknowledge the value of these facts and admit the possibility of a destructive agent other than the oral fluids as a whole. Whatever be this special agent, it is evident that it is formed at the point of attack.

The formation of ammonia in the mouth has already been explained by Dr. Watt in his essays. The initial step in the formation of nitric acid is accepted by all who accept this theory of the cause of dental caries. Those who do not accept this theory must admit the possibility of ammonia being formed in the process of putrefaction whether or not it aids in the formation of nitric acid. Putrefaction is denominated such in distinction from fermentation by the presence of nitrogen. The odor of putrefaction is due to this element. The presence of hydrogen uniting with nitrogen in the proportion of $H_3N$ forms ammonia. Again it is claimed that some bodies are often supercharged with ammonia which may be termed ammonical diathesis, accounting by another means for the presence of ammonia. In this way it is eliminated by the oral mucous membrane and by the gingivæ directly upon the phosphate filling.

The reaction of ammonia upon zinc fillings I believe to be neutralization of the acidity of the fluid portion. The hardening, or let it be crystalization of the zinc mixture, is due to the acidity of the fluid portion; any acid menstrum has this effect upon calcined oxide of zinc. Dilute sulphuric acid produces an uneven quick-setting cement, whereas acetic produces just the opposite results. But for dental purposes phosphoric acid for its intense hardness and chloride of zinc for its therapeutic effect give the best results, yet these acids when neutralized by ammonia have no hardening effect whatever.

When the cement has once hardened the neutralizing effect of ammonia may be objected to. Now I believe the setting of plaster-of-Paris represents what takes place upon mixing calcined oxide of zinc with a dilute acid. In the former the water enters as water of crystallization, in the latter what may be termed an *acid of crystallization.* Now as heat drives off the water of crystallization so does ammonia neutralize the acid of crystallization, and the oxide of zinc falls down as a white powder. These are facts as experiments have shown. Furthermore, this oxide of zinc thus produced is again capable of becoming a hard mass upon union with phosphoric acid and the process may be again repeated. Upon this principle cement instruments may be cleaned by letting them stand a few minutes in ammonia water.

Having accepted that ammonia is formed in the mouth from whatever source and the above being the reaction of this gas upon the crystallized zinc filling material out of the mouth, it is right that we should conclude that the same reaction takes place in the mouth. Litmus tests have shown an alkaline reaction where zinc filling are destroyed by the oral fluids so-called. Ammonia, the product of putrefaction, or ammonia eliminated by the gingivæ, are alike in their effect upon phosphate or oxychloride fillings either as a gas or seven hundred volumes dissolved in one of water, this, I believe, to be the destructive agent found in the oral fluid.

